# *MET* amplification and epithelial-to-mesenchymal transition exist as parallel resistance mechanisms in erlotinib-resistant, *EGFR*-mutated, NSCLC HCC827 cells

**DOI:** 10.1038/oncsis.2017.17

**Published:** 2017-04-03

**Authors:** K R Jakobsen, C Demuth, A T Madsen, D Hussmann, J Vad-Nielsen, A L Nielsen, B S Sorensen

**Affiliations:** 1 Department of Clinical Biochemistry, Aarhus University Hospital, Aarhus, Denmark; 2 Department of Biomedicine, Aarhus University, Aarhus, Denmark

## Abstract

Although many epidermal growth factor receptor (*EGFR*)-mutated lung cancer patients initially benefit from the EGFR-inhibitor erlotinib, all acquire resistance. So far, several mechanisms implicated in resistance have been identified, but the existence of multiple resistance mechanisms in parallel have only been sparsely investigated. In this study, we investigated parallel resistance mechanisms acquired by HCC827, an *EGFR*-mutated adenocarcinoma cell line dependent on EGFR activity and sensitive to erlotinib. The cell line was treated with erlotinib by stepwise escalation of the drug-concentration and erlotinib-resistant (HCC827ER) cells created. HCC827ER cells depicted a mixed epithelial and mesenchymal phenotype. To clarify potential parallel resistance mechanisms, 14 resistant subclones were established by limited dilution. Interestingly, all HCC827ER subclones harbored either a *MET*-amplification (6/14) or underwent EMT (8/14), mechanisms both found in previous studies, but not in co-occurrence. Both subclone-types were resistant to erlotinib, but only *MET*-subclones responded to the MET-inhibitors crizotinib and capmatinib. EMT-subclones on the other hand had markedly increased *FGFR1* expression and responded to the FGFR-inhibitor AZD4547, whereas *MET*-subclones did not. Monitoring gene expression through the development of HCC827ER revealed upregulation of *FGFR1* expression as an early response to erlotinib. In addition, *FGFR1* expression increased upon short-term erlotinib treatment (48 h) identifying a physiological role immediately after erlotinib exposure. The high *FGFR1* expression seen in EMT-subclones was stable even after five passages without erlotinib. Here we show, that parallel resistance mechanisms appear during erlotinib-resistance development in *EGFR*-mutated NSCLC cells and highlight a role for *FGFR1* expression changes as an early response to erlotinib as well as a bypass-signaling mechanism.

## Introduction

Non-small cell lung cancer (NSCLC) is the leading cause of cancer-related death in many developed countries. The 5-year survival rate remains at 10–15% despite advances in treatment options. The poor outcome depicts the advanced disease stage and degree of metastasis at diagnosis, but also the fact that most patients develop resistance to the given treatment and quickly experience progression of their disease. New treatment approaches are based on targeting oncogenic drivers such as epidermal growth factor receptor (EGFR). Erlotinib, a kinase inhibitor targeting EGFR, initially was used successfully as a second-line treatment in NSCLC patients that progressed on standard chemotherapy.^1^ However, it was soon discovered that gefitinib (another EGFR-directed tyrosine kinase inhibitor (TKI)) and erlotinib were especially effective in patients with an activating mutation in *EGFR*.^2, 3, 4^ These patients are now offered EGFR-TKIs as first-line treatment.^5, 6^ Many *EGFR*-mutated patients experience a pronounced initial effect of this treatment, but all acquire resistance over time. Some resistance mechanisms have been discovered including the *T790M* secondary mutation in *EGFR*,^7^ *MET*-amplification,^8^ *FGFR1* overexpression,^9, 10^ *IGF1R* overexpression and gain of cancer stem cell and EMT features.^11^ Inhibitors targeting *MET*,^12^ *FGFR1*^12^ and *T790M*-mutated *EGFR*^13, 14^ are currently under clinical investigation and may facilitate treatment of EGFR-TKI resistance in future settings. However, more than one resistant clone may emerge within the same tumor.^15, 16^ Resistance mechanisms existing in parallel can greatly influence the clinical benefit of treatments targeting only one of the resistant clones. To date, only few studies have investigated the phenomena of co-existing clones in a heterogeneous resistant cell population. In this study, we investigate the parallel emergence of EMT clones dependent on *FGFR1* and clones with *MET*-amplification during erlotinib-resistance development in the *EGFR*-mutated lung cancer cell line HCC827.

## Results

**Figure 1:**
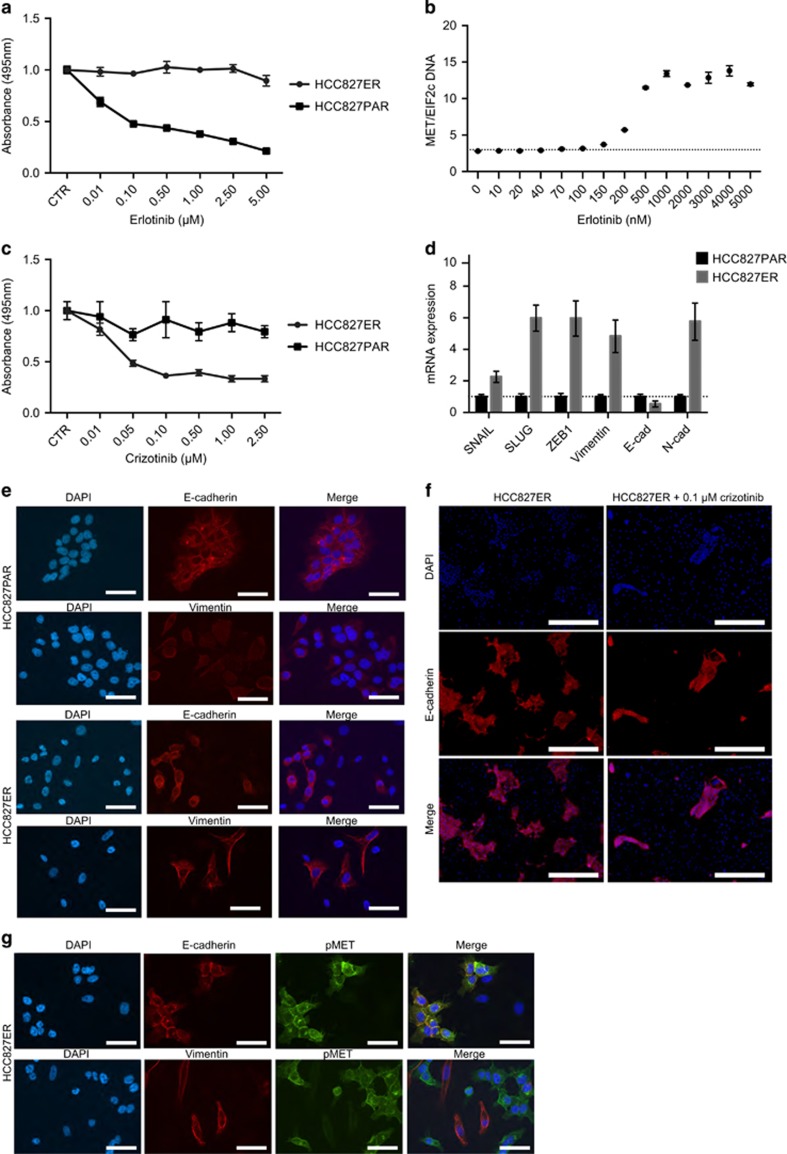
Development of erlotinib-resistant cells. (**a**) MTS assay for erlotinib treatment. All absorbance values were normalized to the absorbance for the control samples of the individual cell line. (**b**) ddPCR analysis of *MET* copy number variation (CNV) during the resistance development. *MET* CNV was normalized to *EIF2c* for each sample. The dotted line represents the *MET* CNV for HCC827PAR. (**c**) MTS assay for crizotinib treatment. All absorbance values were normalized to the absorbance for the control samples of the individual cell line. HCC827ER cells were grown with 5 μm erlotinib in addition to crizotinib. (**d**) qPCR analysis of EMT markers. Expression levels were normalized to *beta-actin* (*ACTB*) and subsequently to the expression in HCC827PAR. (**e**) Immunofluorescence staining of E-cadherin and vimentin (red) in HCC827PAR and ER (× 40, scale bar, 20 μm). (**f**) Immunofluorescence staining of E-cadherin ±0.1 μm crizotinib (× 16, scale bar, 100 μm). (**g**) Immunofluorescence staining of E-cadherin or vimentin (red) and pMET (green; × 40, scale bar, 20 μm). All error bars are s.d.

HCC827 erlotinib-resistant cells (HCC827ER) were established through stepwise escalation of the erlotinib concentration over a period of 4 months. At the halt of the experiment, HCC827ER was no longer responsive to erlotinib at a concentration of 5 μm (Figure 1a). Based on earlier studies reporting *MET* amplification as an EGFR-inhibitor resistance mechanism in HCC827, we investigated the copy number variation of *MET* throughout the course of resistance development. *MET* copy numbers increased when reaching 200 nm erlotinib and remained elevated (Figure 1b). HCC827ER showed increased sensitivity to the MET-inhibitor crizotinib compared with HCC827 parental cells (HCC827PAR) as expected from the *MET-*amplification (Figure 1c).

HCC827ER displayed a distinctive morphology compared with HCC827PAR (Supplementary Figure S1), and the possibility of an EMT-related morphological change was investigated. Increased mRNA expression of mesenchymal markers *SNAIL*, *SLUG*, *ZEB1* and vimentin were observed in HCC827ER as well as a shift from expression of E-cadherin (*E-cad*) to N-cadherin (*N-cad*; Figure 1d). Immunofluorescence staining showed an increased expression of vimentin in some HCC827ER cells, and both E-cadherin-positive and -negative cells were present (Figure 1e). To further investigate the connection between the presence of cells with *MET* amplification and cells with EMT, HCC827ER cells were treated with crizotinib and stained for E-cadherin. Interestingly, the population of E-cadherin-positive cells decreased indicating a link between crizotinib sensitivity and E-cadherin expression in a subpopulation of the cells (Figure 1f). Co-staining of E-cadherin and pMET confirmed an overlap between them (Figure 1g, upper panel). In contrast, co-staining of vimentin and pMET showed little overlap (Figure 1g, lower panel) and hence indicated that *MET-*amplification and EMT had occurred in different subsets of the HCC827ER cells.

### HCC827ER consists of parallel clones

**Figure 2:**
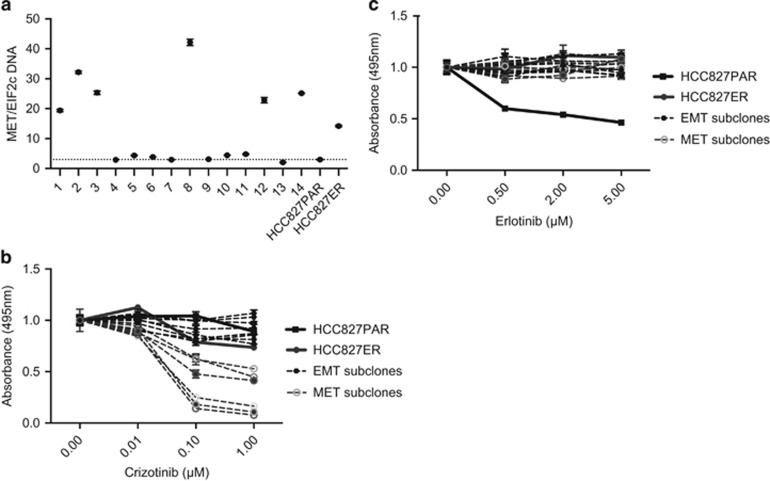
Differential genetic profile and sensitivity to TKI among the HCC827ER subclones. (**a**) The ddPCR analysis of *MET* copy number variation (CNV) of the 14 established subclones. The dotted line represents the CNV of HCC827PAR. (**b**) MTS assay for crizotinib treatment of each subclone. The subclones were divided into three groups according to sensitivity: indifferent (all EMT-subclones, 4–7, 9–11 and 13), intermediary (1, 2 and 14), and highly sensitive (3, 8 and 12). A connection between extent of *MET* CNV and sensitivity was not present. All absorbance values were normalized to the absorbance for the control samples of the individual cell line. EMT- and MET-subclones responded significantly different to crizotinib treatment at 1 μm (*P*<0.0001). (**c**) MTS assay for erlotinib treatment of each subclone. All absorbance values were normalized to the absorbance for the control samples of the individual cell line. HCC827ER and all subclones were grown with 5 μm erlotinib in addition to crizotinib. EMT- and MET-subclones did not respond significantly different to erlotinib treatment at 5 μm (*P*=0.3708).

**Figure 3:**
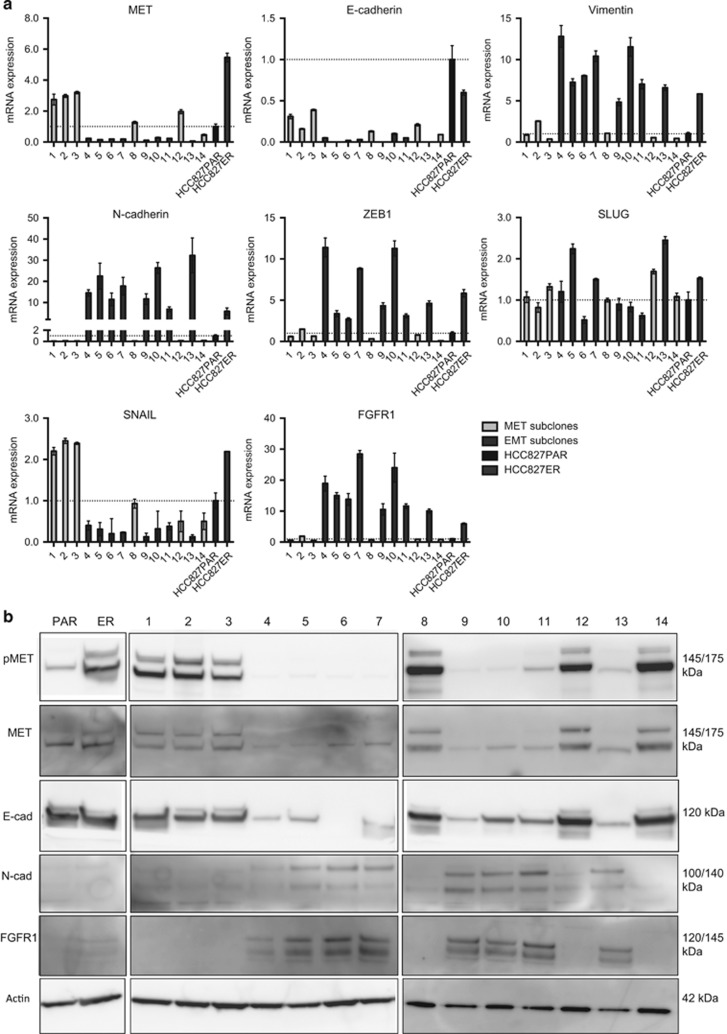
Gene and protein expression patterns in EMT and *MET*-subclones. (**a**) qPCR analysis of *MET* and EMT-marker expression. Expression levels were normalized to *beta-actin* (*ACTB*) and subsequently to the expression in HCC827PAR. EMT- (4–7, 9–11, 13) and *MET*-subclones (1–3, 8, 12, 14) were statistically compared with the following result: *ZEB1* *P*=0.0007, vimentin *P*=0.0007, *SLUG* *P*=0.9291, *SNAIL* *P*=0.0007, *MET* *P*=0.0003, *N-cad* *P*=0.0007, *E-cad* *P*=0.0013, *FGFR1* *P*=0.0007. (**b**) Thirty micrograms of lysate was used for western blot analysis of pMET, MET, E-cadherin and N-cadherin. Beta-actin was used as a loading control. Antibody information is given in Supplementary Table S1.

To gain further insight into the different resistant clones present, 14 subclones of HCC827ER were established using limited dilution. Six of the subclones were *MET*-amplified (Figure 2a) and sensitive to crizotinib (Figure 2b). The remaining eight subclones were not *MET*-amplified, but were equally resistant to erlotinib (Figure 2c). The quantitative PCR (qPCR) analysis and western blotting further confirmed the division of subclones into *MET*-subclones (1–3, 8, 12 and 14) or EMT-subclones (4–7, 9–11 and 13; Figures 3a and b). The expression of EMT markers *SLUG* and *SNAIL* did not follow the pattern of the other EMT-markers and *SNAIL* even had a tendency to be higher expressed in *MET*-subclones (Figure 3a, lower panel). TGF-β-pathway activation is a known inducer of EMT and acquired erlotinib resistance in NSCLC,^17, 18, 19^ but we found no increase in TGF-β1-secretion or activation of SMAD3 in the EMT-subclones (Supplementary Figure S2). On the other hand, *FGFR1* mRNA and protein was expressed in EMT-subclones, but not present in *MET*-subclones (Figures 3a and b). *FGFR1* is known to be able to induce EMT^10, 20^ and was hence investigated further. No expression of *FGFR2* was detected in the cells (data not shown).

**Figure 4:**
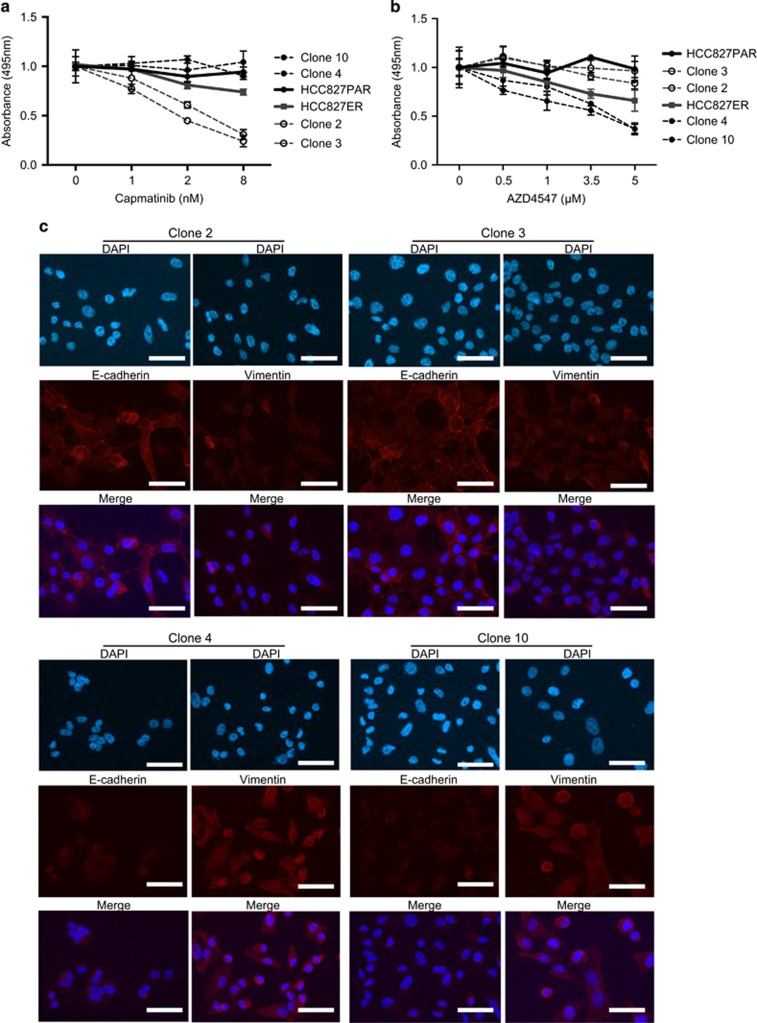
Further characterization of selected EMT- and *MET*-subclones. (**a**) MTS assay for the treatment of selected *MET*-subclones (2+3) and EMT-subclones (4+10) with the MET-inhibitor capmatinib. The cell lines are listed as they appear in the figure. All absorbance values were normalized to the absorbance for the control samples of the individual cell line. EMT- and *MET*-subclones responded significantly different to capmatinib treatment at 8 nm (*P*<0.0121). (**b**) MTS assay for treatment of *MET*-subclones (2+3) and EMT-subclones (4+10) with the FGFR-inhibitor (AZD4547). The cell lines are listed as they appear in the figure. All absorbance values were normalized to the absorbance for the control samples of the individual cell line. EMT- and *MET*-subclones responded significantly different to AZD4547 treatment at 5 μm (*P*<0.0142). (**c**) Immunofluorescence analysis of E-cadherin and vimentin (red) in selected *MET*-subclones (2+3) and EMT-subclones (4+10; × 40, scale bar, 20 μm).

To confirm MET-dependency, and to investigate potential FGFR1-depency, two *MET*-subclones (clone 2 and 3) and two EMT-subclones (clone 4 and 10) were used for further experiments. In these subclones, MET expression status was further confirmed using flow cytometry (Supplementary Figure S3). MET-dependency was confirmed by using an alternative and more specific MET-inhibitor, capmatinib. Only the growth of the two *MET*-subclones, and to some extent the mixed HCC827ER, was inhibited (Figure 4a). On the other hand, the FGFR-inhibitor, AZD4547, was potent in inhibiting the *FGFR1*-expressing EMT-subclones (Figure 4b). The EMT status of the four subclones was confirmed using immunofluorescence staining of E-cadherin and vimentin (Figure 4c).

### Regulation of *FGFR1* expression as a response to erlotinib

**Figure 5:**
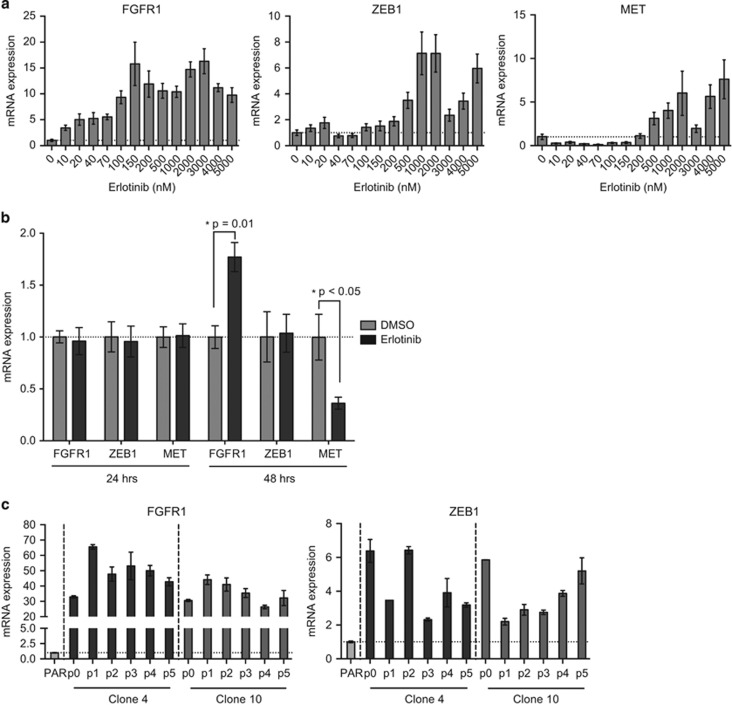
Gene expression as a response to erlotinib. (**a**) *FGFR1*, *ZEB1* and *MET* expression during erlotinib-resistance development. mRNA expression is normalized to *beta-actin* (*ACTB*) and subsequently to the expression in HCC827PAR (0 nm erlotinib) that is given the value 1 (dotted vertical line; error bars=s.d.). (**b**) *FGFR1*, *ZEB1* and *MET* expression after short-term treatment with 5 μm erlotinib. mRNA expression is divided by the expression of the reference gene *beta-actin* (*ACTB*) and the results normalized to the respective dimethyl sulfoxide (DMSO) control (24 or 48 h) that is given the value 1 (dotted vertical line; error bars=s.d.). (**c**) *FGFR1* and *ZEB1* expression upon retrieval of erlotinib in EMT clones. mRNA expression is normalized to *beta-actin* and expression in HCC827PAR (dotted vertical line) p0=with erlotinib, p1–5=number of passages without erlotinib (error bars=s.d.).

Gene expression changes were monitored with qPCR throughout the stepwise escalation of erlotinib concentration (Figure 5a). Interestingly, *FGFR1* expression increased after exposure to 10 nm erlotinib (after approximately 1 week), whereas *ZEB1* and *MET* expression did not change markedly until the cells were exposed to 500 nm (after approximately 7 weeks). A similar increase in *FGFR1* expression was observed in the *EGFR*-mutated NSCLC adenocarcinoma cell line PC-9 during development of erlotinib resistance (data not shown). The same was found upon short time treatment of HCC827PAR with a higher dose of erlotinib (5 μm; Figure 5b). After 24 h, no changes in gene expression were found, whereas *FGFR1* expression increased after 48 h (Figure 5b). *MET* expression on the other hand decreased after 48 h (Figure 5b). Thus, an increase in *FGFR1* expression may be an early response to erlotinib treatment.

As *FGFR1* expression was induced upon erlotinib addition, it may also decrease on erlotinib removal. Hence erlotinib was removed (p0) and clone 4 and 10 were left to grow without erlotinib for five passages (p1–5). In both EMT clones, an initial spike in *FGFR1* expression was observed, after which, the *FGFR1* expression remained high throughout the five passages (Figure 5c, left). Expression of the EMT-marker *ZEB1* fluctuated after erlotinib removal, but also remained elevated compared with HCC827PAR throughout the five passages (Figure 5c, right).

## Discussion

With a growing panel of targeted cancer treatments, there is an augmented need for understanding the mechanisms behind acquired resistance to treatment. However, only a few studies investigate the origin of multiple resistance mechanisms within the same tumor. *MET*-amplification and EMT has previously been described in separate tumors as resistance mechanisms to the EGFR-inhibitor erlotinib.^8, 9, 10^ One previous report has mentioned the co-existence of EMT and *MET-*amplification in gefitinib-resistant HCC827 cells,^21^ but no previous study has reported the EMT phenotype to be associated with *FGFR1* overexpression nor investigated the development and existence of the two mechanisms in parallel. Here, we find that subclones, either dependent on *MET*-amplification or with an EMT phenotype, emerge during the development of erlotinib-resistance in HCC827. In addition, we demonstrate that the EMT-subclones use FGFR1 as a bypass-signaling pathway. EMT has previously been described to be induced through an FGF2–FGFR1 autocrine loop in erlotinib-resistant cells,^9, 10, 20^ which may also be the case in our setting. *MET*-subclones, but not EMT-subclones, respond well to the MET-inhibitors crizotinib and capmatinib. FGFR1-dependent subclones, but not *MET*-subclones, respond well to the FGFR-inhibitor AZD4547. Both MET and FGFR-inhibitors have an effect on the mixed HCC827ER cell line, but less than in the individual subclones. Our finding of the co-existence of two potent, parallel resistance mechanisms highlights the need for more thorough investigation of the possible existence of multiple resistance mechanisms in the individual patient to give a broader spectra treatment.

*FGFR1*-amplification, predominantly found in squamous cell lung carcinoma, is currently an inclusion criterion in a clinical randomized phase I/II study of AZD4547 (NCT01824901). Our study in an adenocarcinoma cell line supports *FGFR1* expression as an additional marker of sensitivity in accordance with previous findings.^22^ Including *FGFR1* expression as a predictive marker for FGFR-inhibitor treatment would expand the pool of patients suitable for treatment, and would include a larger fraction of lung adenocarcinoma patients that rarely exhibit *FGFR1*-amplifications. Hence, further clinical investigation accessing the potential of *FGFR1* expression, as a predictive marker of AZD4547 treatment, is needed.

In clinical settings, it is often only possible to look at molecular changes present in the tumor after it manifests resistance to treatment. Cell models for erlotinib resistance, however, make it possible to monitor changes during the resistance development. Here we identify increased *FGFR1* expression as an early event in erlotinib resistance. Surprisingly, increased *FGFR1* expression precedes both the *MET*-amplification event (Figure 1b) and *ZEB1* expression during EMT (Figure 5a). ZEB1 has previously been described as a mediator of erlotinib resistance,^23^ and it is most likely an important EMT regulator in the described setting owing to overexpression in all EMT-subclones (Figure 3a). Our results point on the early inhibition of FGFR1 as a candidate mechanism to delay or prevent erlotinib-resistance development, but this will clearly need further investigation.

Both FGFR1 and MET pathways constitute alternative survival and growth pathways when the EGFR pathway is inhibited. An increase in *FGFR1* gene expression may be readily obtainable and the cells with increased *FGFR1* expression will have an immediate survival benefit. In addition, increased *FGFR1* signaling can initiate EMT through ZEB1 activation potentiating the drug resistance.^24^ In contrast, *MET*-gene amplification may be a slower event. Either due to *MET*-amplification being a new event that happens as the erlotinib concentration increases or do to gradually selection of already existing cells with *MET*-amplification.

Our study underlines the need for focus on parallel resistance mechanisms in future research on acquired resistance to targeted cancer therapies. This may be pursued both in animal models of resistance as well as from rebiopsies taken at the point of progression, when applicable. In addition, further *in vitro* as well as clinical investigation is needed to access the potential of increased *FGFR1* expression in accordance to erlotinib resistance as well as a predictive marker of AZD4547 treatment.

## Materials and methods

### Cell culture

The lung adenocarcinoma cell line HCC827 was purchased from ATCC/LCG standards (Wesel, Germany) and kept in RPMI 1640 (Gibco, Thermo Fisher Scientific, Waltham, MA, USA) according to the manufacturer’s instructions. The cell samples from before and after resistance development were validated using the Promega GenePrint 10 System according to the manufacturer's instructions (Promega #B9510, Madison, WI, USA). The STR profiles were compared with known ATCC fingerprints (ATCC.org).

### Establishment of erlotinib-resistant HCC827 (HCC827ER)

When initiating the stepwise escalation, 3 million cells were plated in a T75 culture flask with 10 nM erlotinib (Selleckchem, Houston, TX, USA). When the cells reached confluence, half of the flask was harvested for RNA and DNA purification. One fourth of the cells were passaged on with the next drug concentration, and the last fourth was kept as a cells’ stock. When the cells were able to grow with 5 μM erlotinib, the experiment was stopped and the cells were made into stocks for further use. Resistant cells were henceforth kept in 5 μM erlotinib during all experiments unless otherwise noted.

### Establishment of HCC827ER subclones

The established HCC827ER was diluted to approximately 5 cells/ml. One milliliter was seeded in 2 × 24-well dishes. The cells were grown with erlotinib and 14 clones were established. All clones were tested and found to have the initial HCC827 *EGFR* exon19 deletion using the cobas EGFR Mutation Test (Roche Molecular Systems Inc., Branchburg, NJ, USA) on the cobas z 480 analyser according to the supplier’s instructions (http://molecular.roche.com/assays/ Pages/cobasEGFRMutationTest.aspx).

### Genetic analysis

QIAamp DNA mini kit was used for DNA purification from all steps of the resistance development (10–5000 nm) and from all clones. *MET*-amplification status was determined with PrimePCR ddPCR Copy Number Variation Assay (Bio-Rad, Hercules, CA, USA) according to the manufacturer’s instructions. The EIF2C1 assay was used as reference. ddPCR was performed using the QX200 Droplet Digital PCR system (Bio-Rad).

### RNA analysis

#### RNA purification and complementary DNA synthesis

RNA was extracted using the RNeasy mini kit (Qiagen, Hilden, Germany) according to the manufacturer’s instructions. cDNA was synthesized from 100 ng total RNA in a 20 μl reaction mix including 50 μmol/l Oligo(dT), reverse transcriptase (50 units/μl), RNase inhibitors (20 units/μl), 0.4 mmol/l of each dNTP, 1 × PCR buffer, and 25 mmol/l MgCl_2_ (Applied Biosystems, Thermo Fisher Scientific). Reverse transcription was performed on the Perkin-Elmer GeneAmp PCR System 9600 Thermal Cycler (PerkinElmer Inc., Waltham, MA, USA) with the profile: 42 **°**C for 30 min, 99 **°**C for 5 min and 4 **°**C until the samples had cooled. cDNA was stored at **−**20 **°**C until further use.

#### qPCR

Quantitative Real-Time PCR (qPCR) was used to quantify target cDNA with the LightCycler 480(LC480) Real-Time PCR System from Roche (Roche Applied Science, Penzberg, Germany). *Beta-actin* (*ACTB*) was used as a reference gene based on the Normfinder method.^25^ The reaction mix consisted of 5 μl SYBR Green I Master Mix Buffer (Roche Applied Science), 2.5 pmol forward and reverse primer (Eurofins MWG Synthesis GmbH, Ebersberg, Germany), 1 μl cDNA and H_2_0 to a final volume of 10 μl. Primer sequences and annealing temperatures are listed in Supplementary Table S2. qPCR was performed with the following profile: 95 **°**C for 10 min and a total number of 50 cycles with 10 s of melting at 95 **°**C 20 s of annealing at primer-specific temperature, followed by 5 s elongation at 72 **°**C. The concentration was calculated using the standard curve method.

### Western blotting

Protein concentration was determined using the Qubit protein quantitation kit (Thermo Fisher Scientific) and 30 μg protein was loaded on a NuPage 4–12% Bis-Tris gel (Thermo Fisher Scientific). The gel was blotted onto a polyvinylidene fluoride membrane and the membrane was concurrently blocked with either 5% bovine serum albumin or 5% skimmed milk depending on the antibody. The membranes incubated with primary antibody with rotation ON at 4 °C. Hereafter, the membrane was washed and incubated with secondary antibody for 1 h before development with ECL, SuperSignal West Dura Extended Duration Substrate (Thermo Fisher Scientific) using the ImageQuant LAS 4000 system (GE Healthcare Life Sciences, Little Chalfont, UK). For phospho-proteins, the membrane was stripped after ECL detection. The membrane was placed in 10 ml stripping buffer (2% SDS, 62.5 mm Tris-HCl, pH 6.7 in distilled water) mixed with 100 mm β-mercaptoethanol at 55 °C and with rotation of the membrane for 30 min. Hereafter, the membrane was washed 2 × with wash buffer for 10 min before the membrane was blocked and incubated with the corresponding total-protein primary antibody. Antibody information is available in Supplementary Table S1.

### Inhibitor assays

For MTS analysis of drug sensitivity, 5000 cells were plated in each well in a 96-well plate with 200 μl media. Each sample was measured in three replicates including a media control sample. The cells were treated with the indicated inhibitor for 72 h before MTS mixture was added according to the manufacturer’s protocol (CellTiter 96 AQueous Non-Radioactive Cell Proliferation Assay, Promega). For crizotinib, capmatinib and AZD4547 studies, all erlotinib-resistant cell lines were grown in 5 μm erlotinib in addition to the indicated concentration of inhibitor. For the short-term treatment with erlotinib, three RNA samples were harvested for each type of sample. Erlotinib, crizotinib, capmatinib and AZD4547 were obtained through Selleckchem. Each experiment was repeated three times.

### Immunofluorescence analysis

For immunofluorescence analysis, the cells were grown to ~70% confluence on 0.17 mm thick coverslips (Marienfeld, Lauda-Königshofen, Germany), fixed in 4% paraformaldehyde in PBS (Electron Microscopy Sciences, Hatfield, PA, USA) for 20 min at room temperature, and permeabilized in 0.5% Triton X-100 (Sigma-Aldrich, St Louis, MO, USA). The cells were blocked with 1% bovine serum albumin for 1 h and incubated with primary antibody dissolved in blocking buffer for 1 h at room temperature. After washing the cells, they were treated with secondary antibody in blocking buffer for 1 h at room temperature. Cell nuclei were stained with DAPI (Sigma-Aldrich) and the coverslips were mounted with Prolong Gold (Invitrogen, Thermo Fisher Scientific). For immunofluorescence, anti-vimentin (1:500, mouse, Abcam AB20346, Cambridge, UK), anti-E-cadherin (1:1000, mouse, BD Biosciences 610182, San Jose, CA, USA), anti-pMET(1:50, rabbit, Cell Signaling 3129, Danvers, MA, USA) and secondary antibodies Alexa 555 conjugated donkey anti-mouse IgG 1:2000 and Alexa 488 conjugated donkey anti-rabbit IgG 1:2000 (Invitrogen, Thermo Fisher Scientific) were used. A minimum of two coverslips were prepared per sample. All images for immunofluorescence were made on a Zeiss axiovert 200 m microscope (Oberkochen, Germany), with a plan apochromatic objective, a HBO 100 W mercury light source and a CoolSNAP-HQ cooled CCDcamera (Photometrics, Tucson, AZ, USA) operated by MetaMorph (Molecular Devices Corp., Sunnyvale, CA, USA). Color pictures were taken with fixed settings for each series and merged using Image J (National Institutes of Health, Bethesda, MD, USA). No further changes or manipulations were applied to the images.

### Statistics and graphs

Data were interpreted into figures using excel and Graphpad prism (Graphpad Software, La Jolla, CA, USA). Center values represent the mean and error bars s.d. Comparison of statistical significance was performed using an upaired nonparametric *t*-test with a two-sided *P*-value <0.05 considered statistically significant. The groups were tested to show equal variance. In figures displaying individual clones, the statistics were done by grouping and comparing the EMT subgroup with the MET subgroup.

## References

[bib1] Shepherd FA, Pereira JR, Ciuleanu T, Tan EH, Hirsh V, Thongprasert S et al. Erlotinib in previously treated non-small-cell lung cancer. N Engl J Med 2005; 353: 123–132. 16014882 10.1056/NEJMoa050753

[bib2] Lynch TJ, Bell DW, Sordella R, Gurubhagavatula S, Okimoto RA, Brannigan BW et al. Activating mutations in the epidermal growth factor receptor underlying responsiveness of non-small-cell lung cancer to gefitinib. N Engl J Med 2004; 350: 2129–2139. 15118073 10.1056/NEJMoa040938

[bib3] Pao W, Miller V, Zakowski M, Doherty J, Politi K, Sarkaria I et al. EGF receptor gene mutations are common in lung cancers from 'never smokers’ and are associated with sensitivity of tumors to gefitinib and erlotinib. Proc Natl Acad Sci USA 2004; 101: 13306–13311. 15329413 10.1073/pnas.0405220101 PMC516528

[bib4] Paez JG, Jänne PA, Lee JC, Tracy S, Greulich H, Gabriel S et al. EGFR mutations in lung cancer: correlation with clinical response to gefitinib therapy. Science 2004; 304: 1497–1500. 15118125 10.1126/science.1099314

[bib5] Sequist LV, Joshi VA, Jänne PA, Muzikansky A, Fidias P, Meyerson M et al. Response to treatment and survival of patients with non-small cell lung cancer undergoing somatic EGFR mutation testing. Oncologist 2007; 12: 90–98. 17285735 10.1634/theoncologist.12-1-90

[bib6] Weber B, Hager H, Sorensen BS, McCulloch T, Mellemgaard A, Khalil AA et al. EGFR mutation frequency and effectiveness of erlotinib: a prospective observational study in Danish patients with non-small cell lung cancer. Lung Cancer 2014; 83: 224–230. 24388704 10.1016/j.lungcan.2013.11.023

[bib7] Pao W, Miller Va, Politi Ka, Riely GJ, Somwar R, Zakowski MF et al. Acquired resistance of lung adenocarcinomas to gefitinib or erlotinib is associated with a second mutation in the EGFR kinase domain. PLoS Med 2005; 2: e73. 15737014 10.1371/journal.pmed.0020073 PMC549606

[bib8] Engelman Ja, Zejnullahu K, Mitsudomi T, Song Y, Hyland C, Park JO et al. MET amplification leads to gefitinib resistance in lung cancer by activating ERBB3 signaling. Science 2007; 316: 1039–1043. 17463250 10.1126/science.1141478

[bib9] Terai H, Soejima K, Yasuda H, Nakayama S, Hamamoto J, Arai D et al. Activation of the FGF2-FGFR1 autocrine pathway: a novel mechanism of acquired resistance to gefitinib in NSCLC. Mol Cancer Res 2013; 11: 759–767. 23536707 10.1158/1541-7786.MCR-12-0652

[bib10] Ware KE, Hinz TK, Kleczko E, Singleton KR, Marek LA, Helfrich BA et al. A mechanism of resistance to gefitinib mediated by cellular reprogramming and the acquisition of an FGF2-FGFR1 autocrine growth loop. Oncogenesis 2013; 2: e39. 23552882 10.1038/oncsis.2013.4 PMC3641357

[bib11] Shien K, Toyooka S, Yamamoto H, Soh J, Jida M, Thu KL et al. Acquired resistance to EGFR inhibitors is associated with a manifestation of stem cell-like properties in cancer cells. Cancer Res 2013; 73: 3051–3061. 23542356 10.1158/0008-5472.CAN-12-4136 PMC4506773

[bib12] Califano R, Abidin A, Tariq N-U-A, Economopoulou P, Metro G, Mountzios G. Beyond EGFR and ALK inhibition: unravelling and exploiting novel genetic alterations in advanced non small-cell lung cancer. Cancer Treat Rev 2015; 41: 401–411. 25842168 10.1016/j.ctrv.2015.03.009

[bib13] Cross DAE, Ashton SE, Ghiorghiu S, Eberlein C, Nebhan CA, Spitzler PJ et al. AZD9291, an irreversible EGFR TKI, overcomes T790M-mediated resistance to EGFR inhibitors in lung cancer. Cancer Discov 2014; 4: 1046–1061. 24893891 10.1158/2159-8290.CD-14-0337 PMC4315625

[bib14] Sequist LV, Soria J-C, Goldman JW, Wakelee Ha, Gadgeel SM, Varga A et al. Rociletinib in EGFR-mutated non-small-cell lung cancer. N Engl J Med 2015; 372: 1700–1709. 25923550 10.1056/NEJMoa1413654

[bib15] Sequist LV, Waltman BA, Dias-Santagata D, Digumarthy S, Turke AB, Fidias P et al. Genotypic and histological evolution of lung cancers acquiring resistance to EGFR inhibitors. Sci Transl Med 2011; 3: 75ra26. 10.1126/scitranslmed.3002003 PMC3132801 21430269

[bib16] Yu Ha, Arcila ME, Rekhtman N, Sima CS, Zakowski MF, Pao W et al. Analysis of tumor specimens at the time of acquired resistance to EGFR-TKI therapy in 155 patients with EGFR-mutant lung cancers. Clin Cancer Res 2013; 19: 2240–2247. 23470965 10.1158/1078-0432.CCR-12-2246 PMC3630270

[bib17] Kitamura K, Seike M, Okano T, Matsuda K, Miyanaga A, Mizutani H et al. MiR-134/487b/655 cluster regulates TGF-β-induced epithelial-mesenchymal transition and drug resistance to gefitinib by targeting MAGI2 in lung adenocarcinoma cells. Mol Cancer Ther 2013; 13: 444–453. 24258346 10.1158/1535-7163.MCT-13-0448

[bib18] Izumchenko E, Chang X, Michailidi C, Kagohara L, Ravi R, Paz K et al. The TGFβ-miR200-MIG6 pathway orchestrates the EMT-associated kinase switch that induces resistance to EGFR inhibitors. Cancer Res 2014; 74: 3995–4005. 24830724 10.1158/0008-5472.CAN-14-0110 PMC4122100

[bib19] Vazquez-Martin A, Cufí S, Oliveras-Ferraros C, Torres-Garcia VZ, Corominas-Faja B, Cuyàs E et al. IGF-1 R/epithelial-to-mesenchymal transition (EMT) crosstalk suppresses the erlotinib-sensitizing effect of EGFR exon 19 deletion mutations. Sci Rep 2013; 3: 2560. 23994953 10.1038/srep02560 PMC3759044

[bib20] Azuma K, Kawahara A, Sonoda K, Nakashima K, Tashiro K, Watari K et al. FGFR1 activation is an escape mechanism in human lung cancer cells resistant to afatinib, a pan-EGFR family kinase inhibitor. Oncotarget 2014; 5: 5908–5919. 25115383 10.18632/oncotarget.1866 PMC4171601

[bib21] Soucheray M, Capelletti M, Pulido I, Kuang Y, Paweletz CP, Becker JH et al. Intratumoral heterogeneity in EGFR-mutant NSCLC results in divergent resistance mechanisms in response to EGFR tyrosine kinase inhibition. Cancer Res 2015; 75: 4372–4384. 26282169 10.1158/0008-5472.CAN-15-0377 PMC4548796

[bib22] Wynes MW, Hinz TK, Gao D, Martini M, Marek LA, Ware KE et al. FGFR1 mRNA and protein expression, not gene copy number, predict FGFR TKI sensitivity across all lung cancer histologies. Clin Cancer Res 2014; 20: 3299–3309. 24771645 10.1158/1078-0432.CCR-13-3060 PMC4062100

[bib23] Yoshida T, Song L, Bai Y, Kinose F, Li J, Ohaegbulam KC et al. ZEB1 mediates acquired resistance to the epidermal growth factor receptor-tyrosine kinase inhibitors in non-small cell lung cancer. PLoS ONE 2016; 11: e0147344. 26789630 10.1371/journal.pone.0147344 PMC4720447

[bib24] Jakobsen KR, Demuth C, Sorensen BS, Nielsen AL. The role of epithelial to mesenchymal transition in resistance to epidermal growth factor receptor tyrosine kinase inhibitors in non-small cell lung cancer. Transl Lung Cancer Res 2016; 5: 172–182. 27186512 10.21037/tlcr.2016.04.07 PMC4858579

[bib25] Andersen CL, Jensen JL, Ørntoft TF. Normalization of real-time quantitative reverse transcription-PCR data: a model-based variance estimation approach to identify genes suited for normalization, applied to bladder and colon cancer data sets. Cancer Res 2004; 64: 5245–5250. 15289330 10.1158/0008-5472.CAN-04-0496

