# Supplementary material for: MET amplification and epithelial-to-mesenchymal transition exist as parallel resistance mechanisms in erlotinib-resistant, EGFR-mutated, NSCLC HCC827 cells
Source: Oncogenesis. 2017 Apr 3;6(4):e307–. doi: 10.1038/oncsis.2017.17 (PMC5520494; doi:10.1038/oncsis.2017.17)
Supplement: Supplementary Methods [file oncsis201717x1.docx]

**Supplementary methods**

ELISA:

Conditioned media was harvested from 50.000 cells growing in 500 μL 10 % FBS RPMI after 48 hrs. TGF-β1 ELISA was performed using the DuoSet Human TGF-β1 ELISA kit (R&D systems, cat no. DY240-05, Minneapolis, MN, USA) and compared to 10 % FBS RPMI.

Flow cytometry:

Cells were trypsinized, counted and blocked in PBS with 2 % BSA for 15 min on ice. Cells were adjusted to 2*10^6 per mL in PBS + 0.5 %BSA. 1 µL c-MET Alexa Fluor 488 conjugated antibody (Monoclonal Mouse IgG1 Clone # 9510, R&D Systems FAB3582G, Minneapolis, MN, USA) was added per 100 µL cells and incubated for 30 min on ice protected from light. Cells were washed twice with PBS + 0.5% BSA and resuspended in 300 µL PBS + 0.5% BSA. An untreated control was prepared for each cell line. All samples were stained with 3 µL 0.5 mg/mL propidium iodide (PI) (Biolegend, San Diego, CA, USA) prior to analysis on the NovoCyte analyzer (FACS CORE facility at the Department of Biomedicine, Aarhus University). 50,000 events were analyzed per sample. Data analysis was performed with FlowJo® version 10. The experiment was repeated twice.
