# Supplementary material for: MET amplification and epithelial-to-mesenchymal transition exist as parallel resistance mechanisms in erlotinib-resistant, EGFR-mutated, NSCLC HCC827 cells
Source: Oncogenesis. 2017 Apr 3;6(4):e307–. doi: 10.1038/oncsis.2017.17 (PMC5520494; doi:10.1038/oncsis.2017.17)
Supplement: Supplementary Table S1 [file oncsis201717x2.docx]

| **Target** | **Manufactorer** | **Catalog number** | **Host** | **size kDa** | **Antibody diluent** | **Dilution factor** |
| --- | --- | --- | --- | --- | --- | --- |
| **Primary antibodies** |  |  |  |  |  |  |
| Actin | Sigma Aldrich, St. Louis, MO, USA | A5316 | Mouse | 42 | 5% skimmed milk | min. 1:5000 |
| H3 | Cell Signaling, Danvers, MA, USA | 4499S | Rabbit | 17 | 5% skimmed milk | 1:2000 |
| MET | Cell Signaling, Danvers, MA, USA | 3127 | Mouse | 145 (175) | 5% skimmed milk | 1:1000 |
| p-MET | Cell Signaling, Danvers, MA, USA | 3129s | Rabbit | 145 | 5 % BSA | 1:1000 |
| pSMAD3 (s423+s425) | Abcam, Cambridge, UK | ab52903 | Rabbit | 48 | 5 % BSA | 1:1000 |
| t-SMAD3 | Abcam, Cambridge, UK | ab40854 | Rabbit | 48 (55) | 5% skimmed milk | 1:1000 |
| Vimentin | Abcam, Cambridge, UK | AB20346 | Mouse | 54 | 5% skimmed milk | 1:1000 |
| E-cadherin | BD Biosciences, San Jose, CA, USA | 610182 | Mouse | 120 | 5 % BSA | 1:2000 |
| N-cadherin | Abcam, Cambridge, UK | ab76011 | Rabbit | 100 | 5 % BSA | 1:1000 |
| FGFR1 | Cell Signaling, Danvers, MA, USA | 9740S | Rabbit | 92, 120, 145 | 5 % BSA | 1:500 |
| **Secondary antibodies** |  |  |  |  |  |  |
| Anti-rabbit | DAKO, Troy, MI, USA | P0448 | Goat |  | 5% skimmed milk | 1:4000 |
| Anti-mouse | DAKO, Troy, MI, USA | P0447 | Goat |  | 5% skimmed milk | 1:4000 |

**Supplementary Table S1.** Western Blot antibodies
