# Supplementary material for: MET amplification and epithelial-to-mesenchymal transition exist as parallel resistance mechanisms in erlotinib-resistant, EGFR-mutated, NSCLC HCC827 cells
Source: Oncogenesis. 2017 Apr 3;6(4):e307–. doi: 10.1038/oncsis.2017.17 (PMC5520494; doi:10.1038/oncsis.2017.17)
Supplement: Supplementary Table S2 [file oncsis201717x3.docx]

**Supplementary table S2.** qPCR primers

| Gene | Forward Primer | Reverse Primer | Annealing temp | Primer Conc. | Product size |
| --- | --- | --- | --- | --- | --- |
| Beta actin | GGCGCCACCACCATGTACCCT | AGGGGCCGGACTCGTCATACT | 68 | 10 | 202 |
| ZEB1 | AGACATGTGACGCAGTCTGGGT | TGGGCATTCATATGGCTTCTCTCCA | 58 | 5 | 129 |
| Vimentin | GACCAGCTAACCAACGACAAA | TGAAAGATTGCAGGGTGTTT | 58 | 5 | 136 |
| SLUG | GTCCGTCTGCCGCACCTGAG | ACACGGCGGTCCCTACAGCA | 70 | 5 | 72 |
| SNAIL | CGACCACTATGCCGCGCTCT | AGCAGGTGGGCCTGGTCGTA | 68 | 5 | 130 |
| FGFR1 | TGGCCTCCAAGAAGTGCATA | AAATAATGCCTCGGGTGCCA | 60 | 5 | 179 |
| E-cad | GTCCTGGGCAGACTGAATTT | GACCAAGAAATGGATCTGTGG | 58 | 5 | 150 |
| MET | TGGAGACACTGGATGGGAGT | CAGCGCGTTGACTTATTCAT | 60 | 5 | 193 |
| N-cad | CCTGCTTCAGGCGTCTGTAGA | TCATGCACATCCTTCGATAAGACT | 58 | 10 | 102 |
