# Supplementary figures and images for: MET amplification and epithelial-to-mesenchymal transition exist as parallel resistance mechanisms in erlotinib-resistant, EGFR-mutated, NSCLC HCC827 cells
Source: Oncogenesis. 2017 Apr 3;6(4):e307–. doi: 10.1038/oncsis.2017.17 (PMC5520494; doi:10.1038/oncsis.2017.17)

HCC827PAR

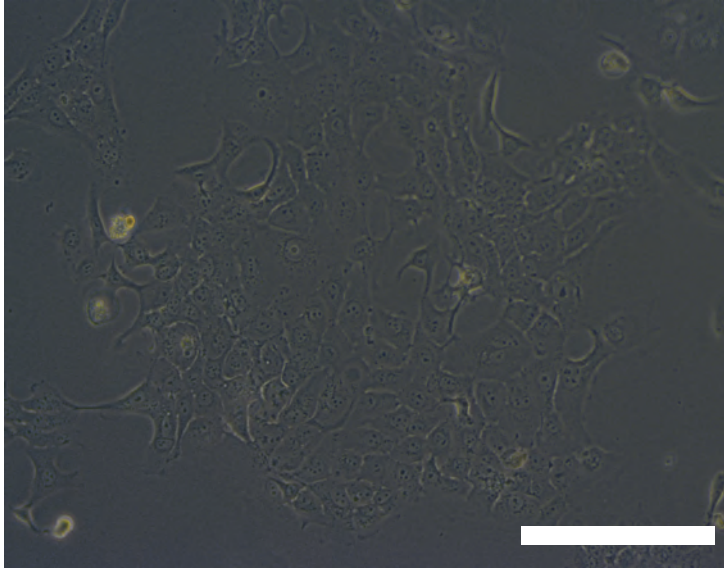

HCC827ER

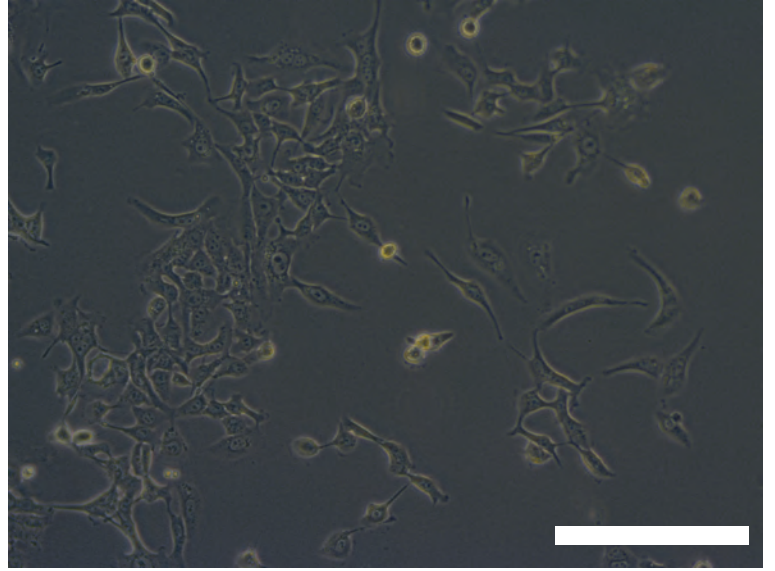

**Supplementary figure S1.** Morphology of HCC827PAR and HCC827ER (x16, size bar = 100  $\mu\text{m}$ ).

Supplement: Supplementary Figure S1 [file oncsis201717x4.pdf]
