# Supplementary material for: MET amplification and epithelial-to-mesenchymal transition exist as parallel resistance mechanisms in erlotinib-resistant, EGFR-mutated, NSCLC HCC827 cells
Source: Oncogenesis. 2017 Apr 3;6(4):e307–. doi: 10.1038/oncsis.2017.17 (PMC5520494; doi:10.1038/oncsis.2017.17)
Supplement: Supplementary Figure S2 [file oncsis201717x5.pdf]

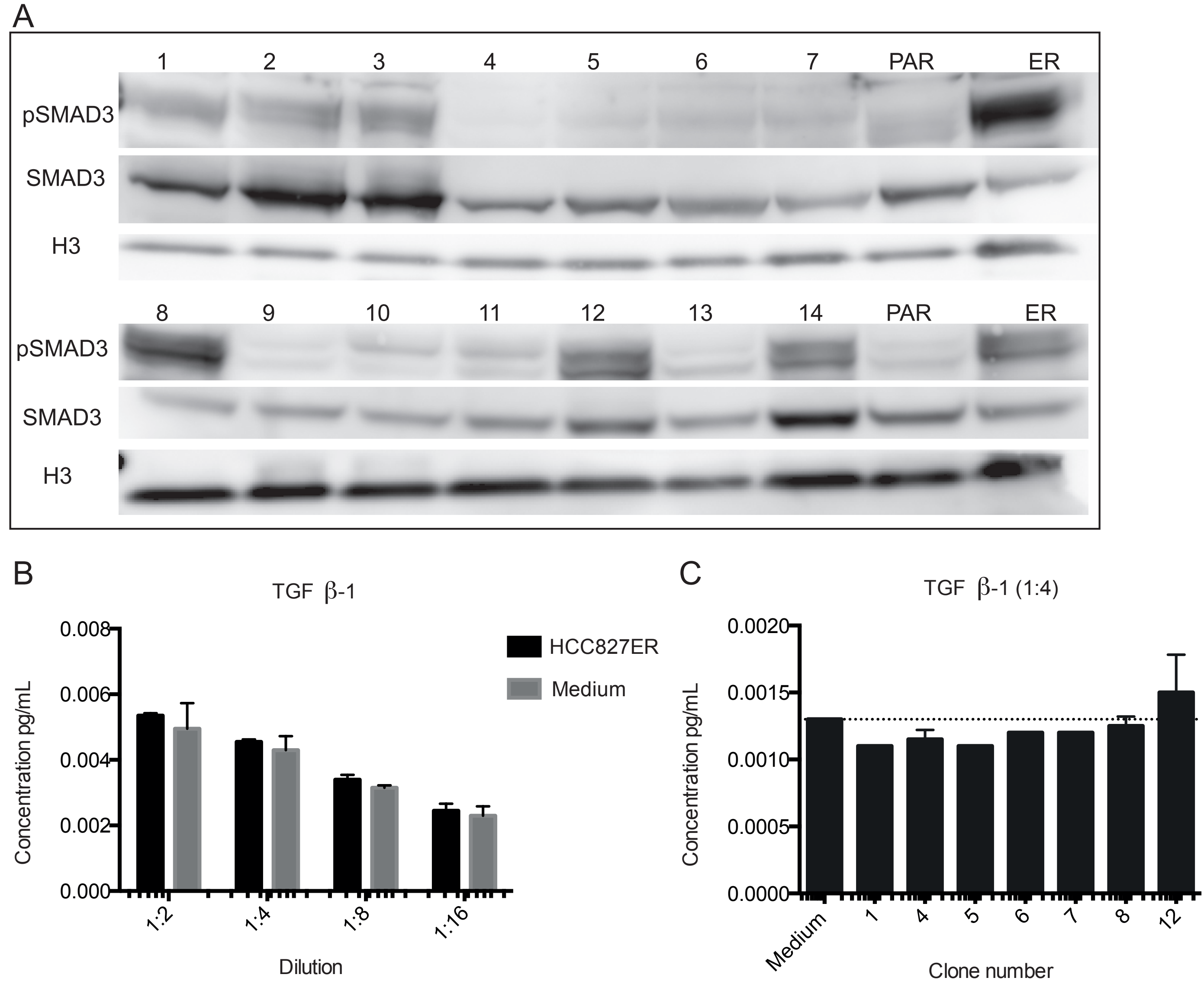

Supplementary figure S2. TGF-β pathway activity. A. Western Blot analysis of phosphorylation of SMAD3 showed activation in MET-dependent subclones (1-3, 8, 12 and 14), but not in EMT-subclones. B. TGF-β secretion was investigated in HCC827ER using ELISA, but there was no significant difference compared to TGF-β levels in growth medium. C. TGF-β secretion was investigated in selected MET-dependent subclones (1, 8, and 12) and EMT-subclones (4-6). None of the subclones showed a significant difference compared to TGF-β levels in growth medium.
