# Supplementary material for: MET amplification and epithelial-to-mesenchymal transition exist as parallel resistance mechanisms in erlotinib-resistant, EGFR-mutated, NSCLC HCC827 cells
Source: Oncogenesis. 2017 Apr 3;6(4):e307–. doi: 10.1038/oncsis.2017.17 (PMC5520494; doi:10.1038/oncsis.2017.17)
Supplement: Supplementary Figure S3 [file oncsis201717x6.pdf]

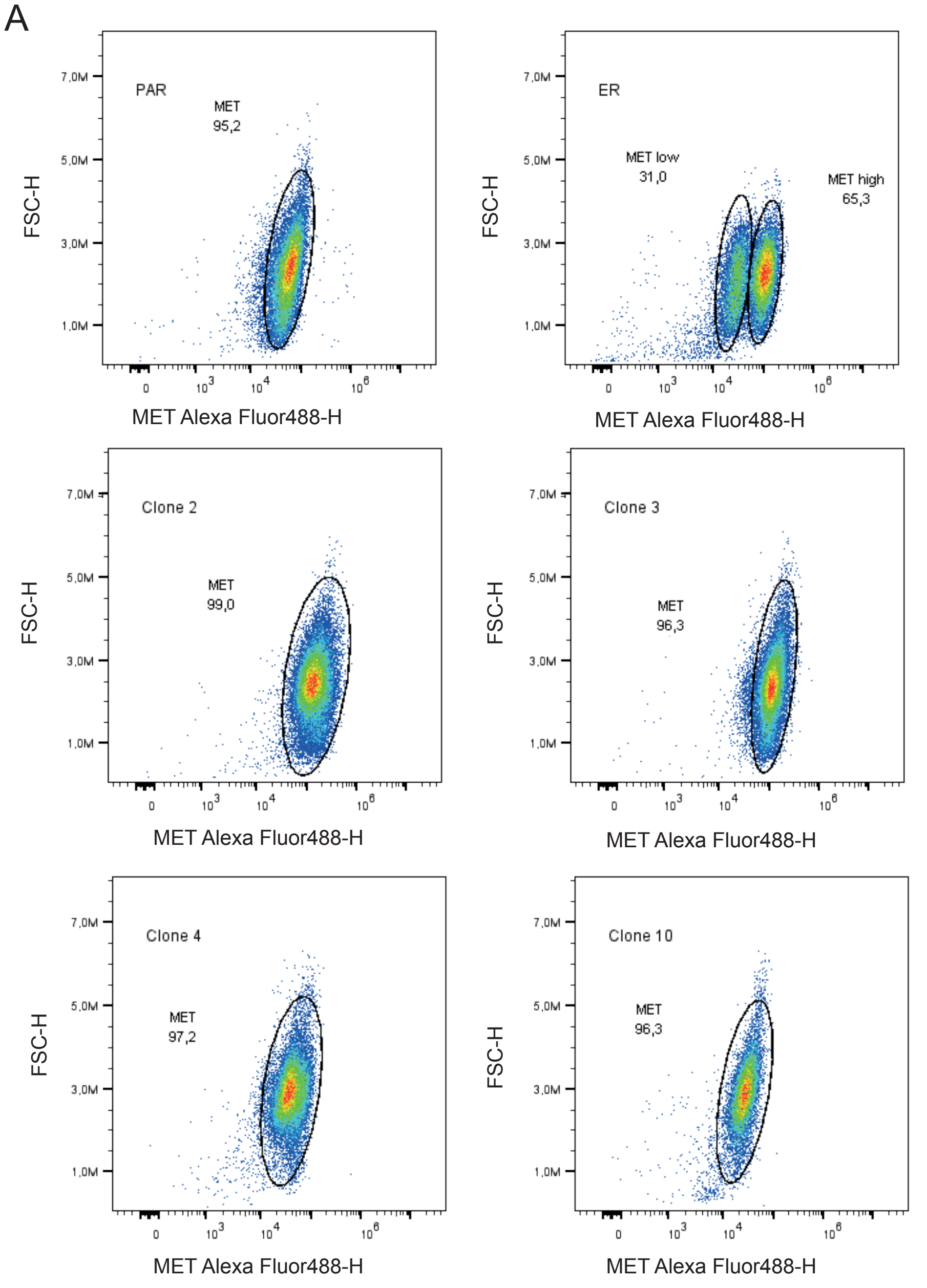

Sample	MFI	SD +/-
HCC827PAR	5,52E+04	2,08E+04
HCC827ER (low)	2,78E+04	9,71E+03
HCC827ER (high)	1,01E+05	2,96E+04
Clone 2	1,34E+05	6,82E+04
Clone 3	1,10E+05	4,03E+04
Clone 4	3,79E+04	1,81E+04
Clone 10	2,57E+04	9,16E+03

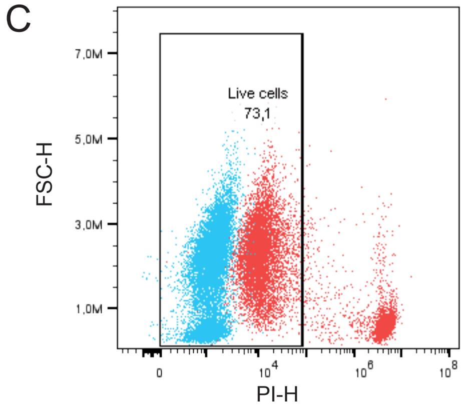

	SampleID	Subset Name	Freq. of Parent
(blue)	PAR unstained	Single Cells	86,2
(red)	PAR	Single Cells	83,6

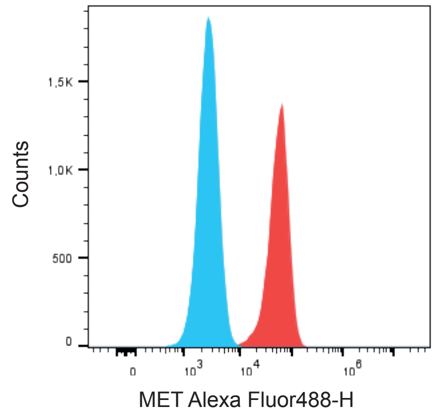

	SampleID	Subset Name	Freq. of Parent
(blue)	PAR unstained	Live cells	99,8
(red)	PAR	Live cells	73,1

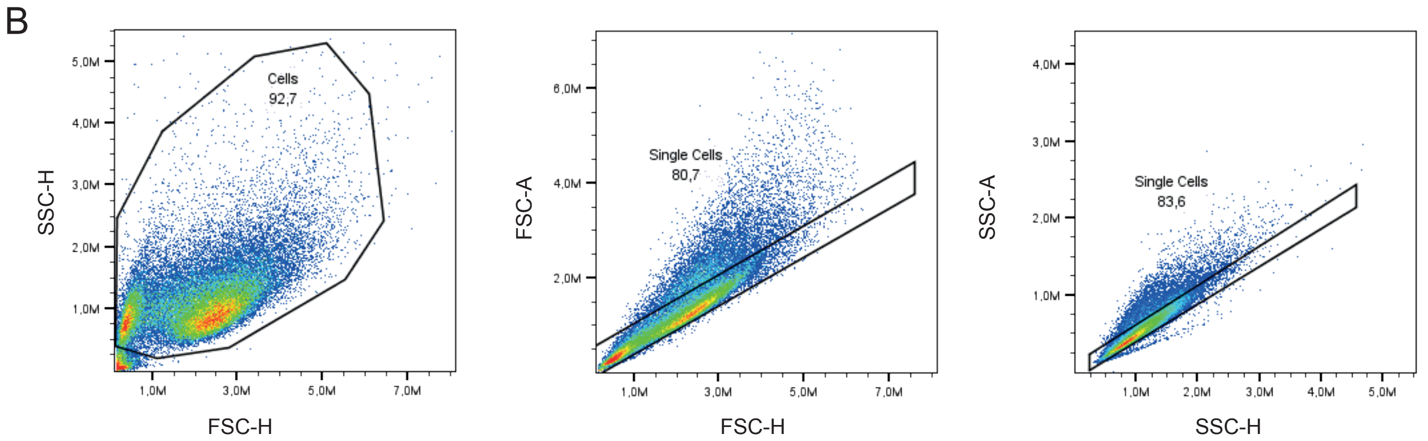

Supplementary figure S3. Flow cytometry analysis of MET expression. A. MET expression in each cell type and Mean Fluorescence Intensity (MFI) for each cell type. HCC827ER divides into two groups, MET low and MET high, corresponding to the MET and the EMT subgroups, as evidenced by similar MFI values for individual MET (clone 2 and 3) and EMT (clone 4 and 10) clones. B. Gating strategy for the experiment. C. MET signal for unstained cells compared with stained cells are showed for HCC827PAR.
